# Increased Seizure Latency and Decreased Severity of Pentylenetetrazol-Induced Seizures in Mice after Essential Oil Administration

**DOI:** 10.1155/2013/532657

**Published:** 2013-06-02

**Authors:** Eleni Koutroumanidou, Athanasios Kimbaris, Alexandros Kortsaris, Eugenia Bezirtzoglou, Moschos Polissiou, Konstantinos Charalabopoulos, Olga Pagonopoulou

**Affiliations:** ^1^Department of Physiology, Medical School, Democritus University of Thrace, Alexandroupolis 68100, Greece; ^2^Faculty of Agricultural Development, Laboratory of Microbiology, Biotechnology and Hygiene, Orestiada 68200, Greece; ^3^Department of Biochemistry, Medical School, Democritus University of Thrace, Alexandroupolis 68100, Greece; ^4^Agricultural University of Athens, Department of Science, Laboratory of Chemistry, 75 Iera Odos Str., Athens GR11855, Greece

## Abstract

The effect of pretreatment with essential oils (EOs) from eight aromatic plants on the seizure latency and severity of pentylenetetrazol- (PTZ-) induced seizures in mice was evaluated. Weight-dependent doses of *Rosmarinus officinalis*, *Ocimum basilicum*, *Mentha spicata*, *Mentha pulegium*, *Lavandula angustifolia*, *Mentha piperita*, *Origanum dictamnus*, and *Origanum vulgare*, isolated from the respective aromatic plants from NE Greece, were administered 60 minutes prior to intraperitoneal (i.p.) injection of a lethal dose of PTZ to eight respective groups of Balb-c mice. Control group received only one i.p. PTZ injection. Motor and behavioral activity of the animals after EOs administration, development of tonic-clonic seizures, seizure latency and severity, and percentage of survival after PTZ administration were determined for each group. All groups of mice treated with the EOs showed reduced activity and stability after the administration of the oil, except for those treated with *O. vulgare* (100% mortality after the administration of the oil). After PTZ administration, mice from the different groups showed increased latency and reduced severity of seizures (ranging from simple twitches to complete seizures). Mice who had received *M. piperita* demonstrated no seizures and 100% survival. The different drastic component and its concentration could account for the diversity of anticonvulsant effects.

## 1. Introduction

Several EOs derived from a variety of plants have been traditionally used as alternative treatments for headaches, migraines, allergies, fatigue, and stress; also, they have long been used as antibacterial agents due to their antimicrobial properties [1].

It is only, in recent years though, that interest has been focused on their possible use as a treatment for diseases of the Central Nervous System (CNS) due to the analgesic, anxiolytic, antidepressant, and antiepileptic properties that they possess. Studies are currently highlighting their potential role in cases of neurological disorders such as epilepsy, stroke, and Alzheimer's disease, [2–6] particularly focusing on their antioxidant as well as anticonvulsant effects [7–9].

Epilepsy is the term used to describe a group of disorders characterized by recurrent spontaneous seizures that apparently result from complex processes involving several neurotransmitter systems such as the glutamatergic, cholinergic, and gabaergic systems [10]. Actual estimations of the prevalence rate for epilepsy are 1-2% of the world population [11], and despite the fact that there is a considerable number of classic and more modern anticonvulsant drugs available for the pharmacological treatment of epilepsy patients worldwide, seizures remain refractory in more than 20% of the cases [10]. Moreover, all current drugs have synthetic origin that causes severe side effects and leads to dependency development [12].

In the wider frame of the ongoing research on the treatment of epilepsy, the present study attempts to evaluate the possible sedative and neuroprotective role of eight EOs isolated from the respective Greek aromatic plants in a PTZ model of epilepsy. The specific aromatic plants were chosen because they are very easy to find as they are widely used in aromatherapy and as culinary herbs making the examination and application of their common properties and potential uses relatively easy. 

## 2. Materials and Methods

### 2.1. Extraction of Essential Oils

Plant samples were collected from the following aromatic plants: *Rosmarinus officinalis*, *Ocimum basilicum*, *Mentha pulegium*, *Mentha spicata*, *Origanum dictamnus*, *Mentha piperita*, *Origanum vulgare*, and *Lavandula angustifolia* from the prefecture of Thrace in northeastern Greece, except for *O. dictamnus* which is endemic in the island of Crete. 

All eight samples were air dried, powdered in an electric blender, and 200 gr of each sample was hydrodistilled. EOs of all tested materials were obtained by the hydrodistillation of the plant samples using a Clevenger type apparatus, for 3 h at 100°C, dried over anhydrous sodium sulphate, filtrated, and stored in a freezer at −20°C until used. 

### 2.2. Gas Chromatography Mass Spectrometry (GC-MS) Analysis

**Figure 1:**
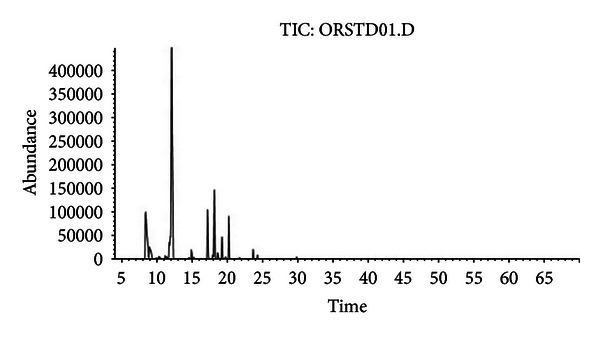
The chromatogram of the essential oil of *Rosmarinus officinalis. *

The EOs were analysed using a Hewlett-Packard II 5890 gas chromatography (GC) system, equipped with a Flame Ionization Detector (FID) and HP-5 ms capillary column (30 m × 0.25 mm, film thickness 0.25 *μ*m) as previously described by Kimbaris et al. [13]. Injector and detector temperatures were set at 220°C and 290°C, respectively. GC oven temperature was programmed to vary between 60°C and 240°C at a rate of 3°C/min and held isothermally for 10 min. Helium was the carrier gas at a flow rate of 1 mL/min. Diluted samples (1/100 in diethyl ether, v/v) of 1.0 *μ*L were injected manually in a splitless mode. Quantitative data were obtained electronically from FID area without the use of correction factors. Qualitative analysis of the EOs was performed using the same conditions as GC and the Hewlett-Packard II 5890 gas chromatograph equipped with Hewlett-Packard II 5972 mass selective detector in the electron impact mode (70 eV) (Figure 1). 

The most abundant ingredient as well as other ingredients of the EOs present at lower levels were identified by comparing GC relative retention times and mass spectra with those of pure standards. Tentative identification of the remaining components was based on the comparison of their mass spectra and elution order with those obtained from the National Institute of Standards and Technology (NIST) 98 and Wiley 275 library data of the GC-MS system and literature [14] (Table 1). 

### 2.3. Animals

Adult female white Balb-c mice weighing approximately 25 gr were used. Animals were housed in cages at room temperature on a 12 : 12 h day/night cycle and given ad libitum access to food and water. On days prior to seizure induction, animals were habituated to handling and the test environment. Animal handling and experiments were performed according to the European guidelines for animal experimentation. 

### 2.4. Essential Oils Administration

Previous studies have shown that EOs penetrate the blood-brain barrier [15]; thus, the i.p. route was chosen for the administration of the EOs. Prior to seizure protocol, animals received an i.p. dose of 1.6 mL/kg of the EOs as described below in the Experimental Procedure. The vehicle employed was a 4 : 1 solution of phosphate-buffered saline and EO. 

### 2.5. The PTZ Model

The PTZ mouse model of generalized myoclonic seizures has long been used for the study of epilepsy in biochemical and pharmacological studies [16]. The prevention of seizures induced by PTZ in laboratory animals is the principal protocol used to characterize a potential anticonvulsant drug [10]. i.p. administration of the drug has dose-dependent results and an injection of 80 mg/kg of body weight is considered to be lethal. Following an i.p. injection of a lethal dose of PTZ there is a typical ten-minute sequence of observations. The first 60 seconds are characterized as latency time and are followed by the first signs of clonic convulsions; these convulsions are associated with movements of the face, mouth, and the front limbs. After that and for two minutes the animal experiences clonic convulsions, followed by tonic clonic seizures lasting for another two minutes; during that phase, extension of the front and back limbs is observed, directed first to the belly and then downwards, a movement that is usually followed by the death of the animal in no more than five minutes [17]. 

According to the findings so far, PTZ seems to act as a factor of generalized stimulation of the CNS, causing cellular excitation [18–21]. The dramatic progression of the epileptic seizures caused by PTZ may be a result of the epileptic activity that begins from the brain stem as this region is found to play an important role in these seizures and is facilitated by the generalized stimulant result of the drug.

### 2.6. Experimental Procedure

Animals were divided into eight groups of six, each one receiving one of the eight EOs tested, plus a ninth control group receiving only the PTZ injection. 1.6 mL/kg of EO were administered i.p. to the animals of the eight groups, and their motor and behavioral activity was recorded per fifteen minutes for an hour. In the completion of the hour, the surviving animals received an i.p. injection of 80 mg/kg PTZ, and the development of tonic clonic seizures, seizure latency, and severity as well as the percentage of survival was determined for each group, compared to the observations from the control group which received only the lethal dose of 80 mg/kg PTZ.

An equal number of animals received the EO only in order to determine the survival of the animals 24 h after the administration of each oil.

The whole procedure as well as the performance of the surviving, after the PTZ injection, animals was video monitored to ensure accurate time measurement and behavioral performance. 

Unpaired Student's *t*-test was performed to compare the results between groups.

## 3. Results

Observation of the motor and behavioral activity of the animals after EOs administration revealed imbalance and reduced movement and in fewer cases lethargy, tensive reactions, and death. Distinctive is the occasion of the essential oil of *O. vulgare* where all animals of the group died after the administration of the oil. After PTZ administration, EO-treated animals demonstrated increased seizure latency, decreased intensity, and differences in the quality of seizures and its characteristics, from simple twitches to complete seizures, always in comparison with the control group. The animals were observed for 24 hours after the completion of the experimental procedure, and the survival percentage for each group was determined.  Table 2 summarizes these observations. 

**Figure 2:**
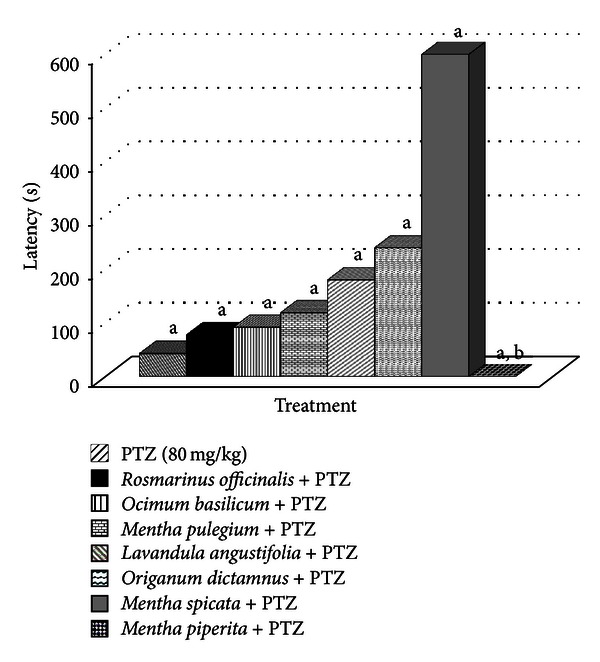
Seizure latency for each essential oil group after PTZ administration. ^a^Differences are statistically significant (*P* > 0.05, Unpaired Student's *t*-test). ^b^Animals of *Mentha piperita* + PTZ group experienced no seizures at all.

In Figure 2 the seizure latency for each EO-treated group after the PTZ administration and for the control group is demonstrated. All EOs presented higher seizure latency for the outbreak of the seizure than the control group for which seizure latency was 43 ± 10 seconds. *M. piperita* EO displayed the best results with no seizures at all. It has to be mentioned that *O. vulgare*-treated animals are not included as all animals of this group died after the injection of the EO as reported above.

All animals receiving only EO survived 24 h after the experiment (except from the *O. vulgare*-treated animals) (data not presented in detail in Table 2).

## 4. Discussion

### 4.1. *M. piperita*-Pretreated Animals (Leading Compounds: Menthol and Menthone)

Previous studies have shown that menthol engages in synergistic excitation of *γ*-aminobutyric acid (GABA) receptors and sodium ion channels resulting in analgesia so it may prove valuable as a leading structure for the synthesis of drugs that target multiple receptors involved with a number of pharmacological effects [22].

The anticonvulsant activity of synthesized menthone compounds has previously been reported in three seizure models in mice which include maximal electroshock seizures (MES), subcutaneous pentylenetetrazol- (scPTZ-) induced seizures, and minimal neurotoxicity test; the compounds were found to elevate GABA levels in the midbrain region, thus indicating that (+/−) 3-menthone semicarbazides could be considered as a lead molecule in designing a potent anticonvulsant drug [23].

On a latest study, (±) 3-menthone aryl acid hydrazone was found to possess better and safer anticonvulsant properties than other reported menthone derivatives including menthone semicarbazides and thiosemicarbazides [24].

In our experimental protocol, *M. piperita* EO was the most effective from all as animals pretreated with it experienced no seizures at all after the administration of PTZ. Furthermore, the survival of animals after the treatment was 100%, showing that it is extremely tolerable by the animals and has the best anticonvulsant results. 

### 4.2. *M. spicata*-Pretreated Animals (Leading Compounds: Piperitone Epoxide and Piperitenone Epoxide)

Trans-piperitone epoxide was found in a percentage of 9% in the analysis of the EO of *Mentha longifolia* [25] while cis-piperitone epoxide was found in a percentage of 0.8–3.3% in a study of the composition of the EO of *Origanum tyttanthum* [26].

No studies dealing with the anticonvulsant activity of those two epoxides have been contacted yet; therefore, our study is the first one demonstrating a possible anticonvulsant effect of these substances. However, the low percentage of survival of the animals after the treatment should be an aversive factor for the use of this EO as an anticonvulsant. Perhaps with the administration of a lower dose the survival could be higher. 

### 4.3. *O. dictamnus* (Leading Compounds: Carvacrol and P-Cymene)

Antimicrobial and antifungal—due mainly to carvacrol—properties of *O. dictamnus *have been described since ancient years but have also been shown experimentally in recent studies [1]. Antiepileptic effects of the main constituents of the seeds of another plant, *Nigella sativa*, one of its main components being p-cymene, were investigated using PTZ- and (MES-) induced convulsions [27]. All of the seed constituents protected mice effectively against PTZ-induced convulsions; the activity of the volatile oil in this model may be attributed mainly to its content of thymoquinone and p-cymene [27].

In our experiments, animals who received *O. dictamnus* EO prior to PTZ showed statistically significant increase of seizure latency, as well as 100% percentage of survival after the administration of the treatment, indicating a possible protective effect. 

### 4.4. *L. angustifolia* and *O. basilicum* (Leading Compound: Linalool)

Following i.p. administration in mice, linalool produces antinociceptive and antihyperalgesic effects on different animal models in addition to anti-inflammatory properties; linalool also possesses anticonvulsant activity in experimental models of epilepsy [28].

Psychopharmacological *in vivo* evaluation of linalool showed that this compound has dose-dependent marked sedative effects at the CNS including hypnotic, anticonvulsant and hypothermic properties; an inhibitory effect of linalool on glutamate binding in rat cortex was reported, suggesting that this neurochemical effect might be underlying linalool psychopharmacological effects [29].

Elisabetsky et al. (1999) [30] examined the pharmacodynamic basis of the previously-established anticonvulsant properties of linalool and the effects of this compound on behavioral and neurochemical aspects of glutamate expression in experimental seizure models. Data indicated that linalool modulates glutamate activation expression *in vitro* and *in vivo*, and it also partially inhibited and significantly delayed the behavioral expression of PTZ kindling. 

An inhibitory effect of linalool on the acetylcholine (ACh) release and on the channel open time in the mouse neuromuscular junction has been reported; more specifically, linalool induced a reduction of the ACh-evoked release, which could be ascribed to some interaction with presynaptic function [31].

To further clarify the anticonvulsive mechanisms of linalool, the effects of linalool on binding of *N*-methyl-D-aspartate (NMDA) glutamate antagonist and GABA_A_ agonist to mouse cortical membranes were studied by Silva Brum et al. (2001) [32], and data suggested that the anticonvulsant mode of action of linalool includes a direct interaction with the NMDA receptor complex. Considering the profile of sedative and anticonvulsant central effects of linalool in various mouse models and the alleged effects of inhaled lavender EO, the sedative effects of inhaled linalool in mice were examined, and it was concluded that linalool inhaled for one hour seems to induce sedation without significant impairment in motor abilities, a side effect shared by most psycholeptic drugs [33].

In accordance to the above data, animals who received *L. angustifolia* EO in our experiments prior to PTZ showed statistically significant increase of seizure latency as well as the above average (66.6%) percentage of survival, indicating a possible protective effect. 

### 4.5. *M. pulegium* (Leading Compound: Pulegone)

*Calamintha officinalis Moench* EO which also contains pulegone provides protection against PTZ-induced convulsions [34]. Previous study data suggest that (R)-(+)-pulegone is a psychoactive compound and has the profile of an analgesic drug; (+)-pulegone also significantly increases the latency of convulsions as assessed by the PTZ method [35].

In our experiments and in accordance with the abovementioned data, *M. pulegium* EO was highly tolerable since 86% of the animals survived after the treatment; also, animals exerted increased time of seizure latency.

### 4.6. *R. officinalis* (Leading Compound: Eucalyptol a.k.a. 1,8-cineole)

1,8-cineole has been found in the EO *Calamintha officinalis Moench* in a percentage of 6.4% [34]. ln rodents, it produces the typical effects in behavior of a nonselective CNS-depressant drug; it potentiates the hypnotic effects of sodium pentobarbital, decreasing the induction time and enhancing the sleeping time. Moreover, it produces a decrease in body temperature and a protection against PTZ-induced convulsions [34].

The composition analysis of the EO of *Ugni myricoides* showed that it contains 1,8-cineole in a percentage of 11.9%; this orally administered oil was found to possess systemic antihypernociceptive properties in inflammatory and neuropathic models of hypernociception in mice [36].

In our experiments, animals who received *R. officinalis* EO prior to PTZ showed a rather small but statistically significant increase of seizure latency as well as the above average (60%) percentage of survival after the administration of the EO and PTZ treatment. We could possibly claim that these results attribute to the existing data as for the possible protective effect of the EO of *R. officinalis.*

## 5. Conclusions

The administration of the tested EOs delivered different results to the PTZ-induced seizures. *M. piperita* EO gave very encouraging results as, despite that the PTZ dose was considered lethal, all animals of the specific group managed to survive for 24 hours after the injection without experiencing any seizures at all. It is possible that the cause of this different antiepileptic action is the dominant drastic component and the concentration in which it is found in the oil. 

Further research including the administration of the pure leading compound of the EO as well as in some cases lowering the dose of the EO will elucidate the role that it may play in the development of PTZ-induced seizures thus providing possible anticonvulsant agents of a more natural origin. 

## Figures and Tables

**Table 1:** Essential oils relative percentage compositions.

Main components^a^	Essential oils							
	*Rosmarinus officinalis *	*Ocimum basilicum *	*Mentha pulegium *	*Mentha spicata *	*Lavandula angustifolia *	*Mentha piperita *	*Origanum dictamnus *	*Origanum vulgare *
**α**-Pinene^b^	14.3							
*p*-Cymene^b^							23.1	9.5
Eucalyptol^b^	44.9	7.3			6.0			
Linalool^b^		56.5			27.2			
Campho^b^					6.5			
Menthone^b^						21.9		
Menthone *(iso)* ^b^						10.4		
Borneol^b^	9.3							
Menthol^b^			13.0			27.7		
Pulegone^b^			62.0					
Piperitone epoxide^c^				23.8				
Linal*οο*l acetate^c^					18.0			
Carvacrol^b^							43.9	79.0
Piperitenone Epoxide^c^				41.0				
Oil yield^d^	2.2	1.6	2.3	2.1	1.7	1.2	2.4	4.1

^a^Compounds listed in order of elution from an HP-5 MS column. ^b^Comparison with pure standards. ^c^Tentative identification based on data obtained from NIST98 and Wiley 275 Library of the GC-MS system and literature data. ^d^Yield of isolated oils was expressed as mL of essential oil/100 gr of dry material.

**Table 2:** Animal survival percentages (at 24 h) and seizure latency times after the administration of the essential oils and PTZ.

Essential oil	% survival	Seizure latency (in sec)
*Mentha piperita*	100%	No seizures at all
*Origanum dictamnus*	100%	240 ± 15
*Mentha pulegium *	86%	119 ± 20
*Ocimum basilicum *	80%	92 ± 12
*Lavandula angustifolia *	66.6%	180 ± 19
*Rosmarinus officinalis *	60%	78 ± 8
*Mentha spicata *	33.3%	600 ± 31
